# The Effect of The Release of Exogenous Nitric Oxide on The Responses of The Pregnant Human Myometrium To Oxytocin*

**DOI:** 10.34763/devperiodmed.20182204.301307

**Published:** 2019-01-14

**Authors:** Beata Modzelewska, Tomasz Kleszczewski, Anna Kostrzewska

**Affiliations:** 1 Departments of Biophysics, Medical University of Białystok, Białystok, Poland; 2 Łomża Medical College of the Universal Educational Society, Łomża, Poland

**Keywords:** oxytocin, a nitric oxide donor, pregnant myometrium, uterus contractions, preterm labor, oxytocin, a nitric oxide donor, pregnant myometrium, uterus contractions, preterm labor

## Abstract

*Currently there is insufficient evidence to support the routine administration of nitric oxide donors in the treatment of threatened preterm labor. An understanding of the role that nitric oxide plays in the management of threatened preterm labor may lead to more effective treatment and prevention. This is why the aim of our study was to examine the involvement of exogenous nitric oxide release in regulating responses of the human pregnant myometrium to oxytocin. Biopsies of human myometrial tissue during pregnancy were obtained from 8 pregnant women, aged 21-35 years. The responses of the specimens to oxytocin in the absence and presence of a DETA/NO were recorded under isometric conditions. Preincubation with exogenous nitric oxide significantly (p<0.001) attenuated the contractile response of the uterine strips to oxytocin in concentrations higher than 10*^-8^ *mol/L. The inhibition of nitric oxide synthesis alone or in combination with DETA/NO incubation did not significantly change the oxytocin contractile effect in the concentration-response curve. Moreover, there was no significant variation in the mean value for log EC*_50_ *for oxytocin between the group with oxytocin alone and other groups. We present evidence in support of the hypothesis that continuous nitric oxide supply to the human pregnant myometrium environment attenuates its response to oxytocin but only when endogenous production of nitric oxide is not impaired*.

## Streszczenie

*Przy obecnym stanie wiedzy nie ma wystarczających przesłanek do rutynowego podawania donorów tlenku azotu w leczeniu zagrażającego porodu przedwczesnego. Zrozumienie roli tlenku azotu jako jednego z czynników utrzymujących mięsień macicy w stanie niepobudzonym podczas ciąży może prowadzić do skuteczniejszego leczenia i zapobiegania przedwczesnego zakończenia ciąży. Dlatego celem naszego badania było zbadanie udziału egzogennego uwalniania tlenku azotu tlenku azotu w regulowaniu reakcji ludzkiego myometrium na oksytocynę. Biopsje tkanki mięśniowej macicy uzyskano od 8 ciężarnych kobiet w wieku 21-35 lat. Reakcje skrawków mięśniowych na oksytocynę bez i w obecności DETA\NO rejestrowano w warunkach izometrycznych. Preinkubacja z egzogennym tlenkiem azotu istotnie (p<0,001) osłabiała reakcję skurczową badanych skrawków macicy na oksytocynę w stężeniach powyżej 10*^-8^ *mol/L. Wcześniejsze hamowanie syntezy tlenku azotu lub w kombinacji z inkubacją z DETA/NO nie zmieniło znacząco efektu działania oksytocyny w krzywej dawka-efekt. Co więcej, nie stwierdzono istotnej zmiany średniej wartości log EC*_50_ *dla oksytocyny pomiędzy grupą z samą oksytocyną, a innymi grupami badawczymi. W przedstawianej pracy prezentujemy dane na poparcie hipotezy, że długotrwałe dostarczanie tlenku azotu do myometrium osłabia jego reakcję na oksytocynę, ale tylko wtedy, gdy endogenna produkcja tlenku azotu nie jest osłabiona*.

## Introduction

Preterm labor is defined as the occurrence of regular painful uterine contractions of sufficient frequency and intensity to effect the progressive effacement and dilation of the cervix culminating in delivery of a preterm infant before 37 weeks of pregnancy [1]. Increased uterine contractility at term and preterm labor results from several mechanisms, one of which is the activation and stimulation of the myometrium by increasing the concentration of oxytocin (OXT) [2]. Therefore, uterine contractions are induced *via* the activation of phospholipase C and release of inositol 1,4,5-triphosphate, 1,2-diacylglycerol, and intracellular calcium [2].

OXT mediates its biological effects by acting on specific receptors (OXTR) [3] but to some extent it also activates the myometrium via the vasopressin V_1a_ receptor [4]. The pregnant uterus is one of the traditional targets of OXT and is one of the most potent uterotonic agents clinically used to induce labor [5].

Nitric oxide (NO) is believed to participate in maintaining relaxation of the myometrium quiescence throughout gestation [6]. Therefore, decreased production of NO as well as decreased myometrial response in late pregnancy is supposed to promote labor [7, 8]. Functional experiments *in vitro* have reported that nitric oxide (NO) donors attenuate myometrial contractility [9]. To stimulate a preeclampsia-like syndrome in rats and mice NO production was blocked by administering a NOS-inhibiting agent, which implied a potential decrease in NO production and NOS activity [10]. However, several researchers disagree as to the importance of NO in preeclampsia. Some of them have suggested the possible primary or secondary role of NO in the development of preeclampsia, but few of them have associated the dysfunction of NO synthesis with other metabolic disorders described in this condition [11].

At present, there is insufficient evidence to support the routine administration of NO donors to the treatment of threatened preterm labor. Nevertheless, several studies have demonstrated the beneficial role of nitric oxide agents, in particular glyceryl trinitrate and L-arginine in lowering blood pressure and improving uteroplacental blood flow velocities [12]. Much research still needs to be done. Understanding the role of NO in the management of threatened preterm labor may lead to more effective treatment and prevention. Therefore, the aim of our study was to investigate the contribution of exogenous NO in the regulation of responses of the human pregnant myometrium to OXT.

## Methods

### Human myometrial tissue

This study was carried out in accordance with the principles of the World Medical Association’s Declaration of Helsinki, the International Conference on Harmonisation Guideline for Good Clinical Practice, and the laws and regulations of Poland. Biopsies of human myometrial tissue during pregnancy were obtained at elective caesarean section operations from 8 pregnant women, aged 21–35 years. All the women, who had previously qualified for caesarean section surgery, were fully informed about the nature and procedure of the study and gave their written consent to participation. The Bioethics Committee of the Medical University of Białystok had earlier approved its protocol (Opinion No. R-I-002/109/2010).

### Sample processing and data acquisition

The biopsies were excised from the upper lip of the lower uterine segment, incision in the midline, i.e. the upper portion of the lower uterine segment. Immediately upon collection, the samples were placed in ice-cold Tyrode’s solution and transferred to the laboratory where they were processed as previously described [13]. The strips were then mounted in an isolated organ bath containing 20 ml of Tyrode’s solution thermostatically maintained at 37^o^C, pH 7.4, and bubbled with carbogen (95%O_2_ + 5%CO_2_). The strips were left for an equilibration period of 1-2 hours, within which the passive tension was adjusted to 2 mN. After equilibration, regular phasic contractions were achieved.

Myometrium activity was recorded by a force transducer with digital output (BIO-SYS-TECH, Bialystok, Poland) and with the DASYLab software unit (version 9.0; Laboratory Data Acquisition System, SuperLogics, Waltham, MA, USA). Before each experiment, the strips were activated by 80 mmol/L K^+^. Appropriate controls were run under similar experimental conditions in strips of uterus obtained from the same woman. Only one dose-response curve was performed on each preparation.

Diethylenetriamine/nitric oxide (DETA/NO) – a spontaneously releasing nitric oxide donor at the concentration of 10^-4^ mol/L was added to the organ bath in a series of experiments. To estimate the prolonged involvement of NO, spontaneous contraction recording (20 min) and again after 20 hours were run under similar organ bath conditions obtained from the same patient. In the current study, 20 min preincubation with L-arginine analog NG -nitro-L-arginine (L-NNA) (3·10^-4^ mol/L) was performed to inhibit endogenous NO production [14].

Oxytocin (OXT) was added cumulatively to the organ chambers (range 10^-14^–10^-6^ mol/L) at 10-minute intervals, and the effects were recorded. On the basis of the results obtained, incubation with 10^-4^ mol/L DETA/NO with or without previous NOS inhibition (by L-NNA) was performed. Then the observation of the OXT effects on uterine contractions was conducted during continuous decomposition of DETA/NO.

The responses were quantified by the area under the curve (AUC). The AUC reflected the total quantity of changes in amplitude, frequency of active contractions, and basal tension of myometrial strips over time before and after the administration of the given drug. The AUC was measured as the area under all recorded contractions over a 10-minute interval before the addition of the substances that were tested [15].

### Drugs and solutions

Drugs and reagents were purchased from Sigma-Aldrich (St. Louis, MO, USA): oxytocin (OXT, oxytocic hormone); 2,2’-(hydroxynitrosohydrazono)bis-ethanimine (diethylenetriamine/nitric oxide, DETA/NO); N5-(nitroamidino)-L-2,5-diaminopentanoic acid, NG-NO2-L-Arg (N_ω_-nitro-L-arginine, L-NNA).

A stock solution of OXT was prepared daily using bidistilled water. A series of dilutions were prepared on the day of the experiment. All the substances were added directly to the organ bath containing a Tyrode solution composed of (mM): NaCl 136.9; KCl 2.70; MgCl_2_ 1.05; NaH_2_PO4 1.33; CaCl_2_ 1.80; NaHCO_3_ 25.0; and glucose 5.0. which was also made on a daily basis.

### Measurements of contraction parameters

The contractile activity of the myometrial strips before the administration of the substances tested was treated as a control (set as 100%). The AUC was evaluated by calculating the integral of the appropriate section of the curve. Concentration-response curves were fitted to the logistic equation using nonlinear regression (PRISM 6.0, Graph Pad Software Inc., San Diego, CA, USA). The maximal response (E_max_) was expressed as a percentage of the contractile activity before the administration of the substances tested, whereas the concentrations of agents that resulted in a half-maximal effect were expressed as -log EC_50_.

### Statistical analysis

All the results were expressed as means ± SEM with “N” denoting the number of experiments performed on myometrial strips from different patients. Dose-response was determined using analysis of variance (ANOVA) followed by a non-parametric or parametric Dunnett’s multiple comparison test where appropriate. All the analyses were performed using Prism 6 for Windows (version 6.0, GraphPad Software Inc., San Diego, CA). Values were considered to be statistically significant at p<0.05.

## Results

**Fig. 1:**
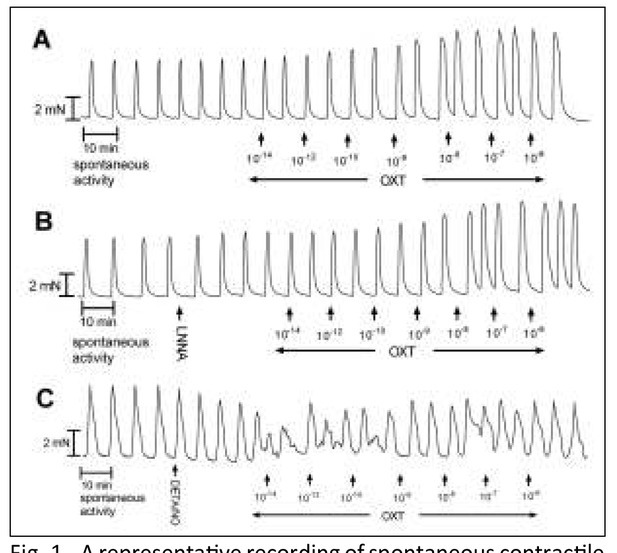
A representative recording of spontaneous contractile activity of the myometrial strips and the effect of cumulatively administered OXT (10^-14^– 10^-6^ mol/L): (A) without any blocker and (B) after preincubation with L-NNA (3·10^-4^ mol/L) and (C) incubation with DETA/NO (10^-4^ mol/L).

Spontaneous phase contractile activity was developed in 75.00% of the myometrium strips obtained from pregnant women (Fig. 1). All the strips examined presented a similar basal tension, mean 1.97±0.82 mN (N=6). The mean amplitude was 4.68±0.42 mN and the mean frequency of spontaneous contractions for 10 min was 1.72±0.31.

### Effects of LNNA

Inhibition of NOS by 3·10^-4^ mol/L L-NNA caused an intensification of the spontaneous contractile activity of myometrial strips demonstrated as a significant increase of the AUC 115.5±2.11% (Fig. 1B). This effect is similar to that shown in other tissues [14,16] and in like manner involved a significant increase in the basal tension, the mean frequency and amplitude of contractions.

### Effects of DETA/NO

**Fig. 2:**
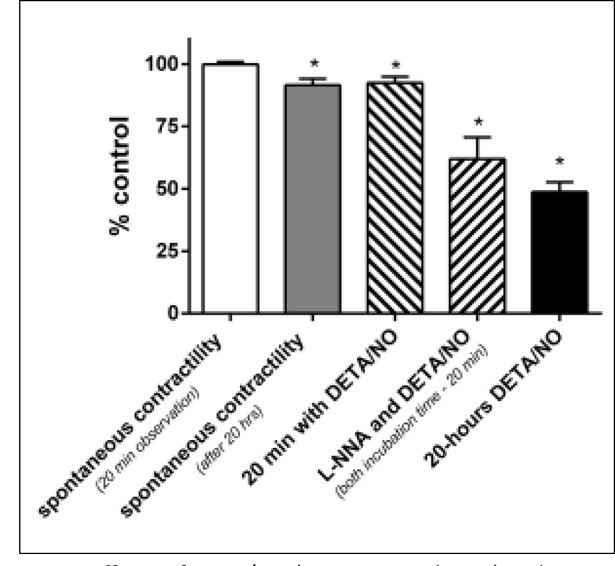
Effects of DETA/NO (20 min or 20 hours) and L-NNA (20 min) incubation on the pregnant myometrium, as measured by the area under the curve. Spontaneous contractile activity after 20 min observation was treated as control. *- p<0.05.

To examine the influence of the prolonged effects of exogenous NO release on uterine contractility, we observed it after 20 min (Fig. 2) or 20-hour incubation with 10^-4^ mol/L DETA/NO. DETA/NO has been shown to release nitric oxide with an estimated half-life of about 20 hours in a solution with pH 7.4 at 37° C [17]. In the current studies, this compound produced a substantial inhibition of spontaneous contractility after 20 min and considerably stronger after 20-hour incubation of myometrial strips (92.45±2.44% and 63.74±4.58%, respectively, p<0.001). After 20-hour incubation, the AUC of the control pregnant strips changed significantly (91.54±2.52%, p<0.05). When we examined the effect of 20-min incubation with DETA/NO after inhibiting endogenous NO production, the decrease was 62.99±7.23% (p<0.01) of AUC (Fig. 2).

To evaluate the effect of DETA/NO on the OXT-induced maximum response of myometrial smooth muscle, we conducted experiments without and after incubation with L-NNA. One of the doubtless results of the tests was the significant inhibition of contractile activity of the strips that were used. The weakening of contractions was deeper after 30 min than after 10 min of incubation with DETA/NO (45.39%±7.78, N=7; 76.52%±7.55, N=6, respectively, p< 0.05). A similar trend, but insignificant, occurred when NOS production was inhibited, after 30 min of DETA/NO incubationic 31.19%±8.43, N=7 and after 10 min 46.72%±6.79, N=7. The attenuation in AUC value was considerably deeper after 10 min of preincubation with the NO donor, when NOS was additionally inhibited (p<0.05). No significant changes were observed after 30 minutes of incubation.

### Contractile responses to oxytocin

**Fig. 3:**
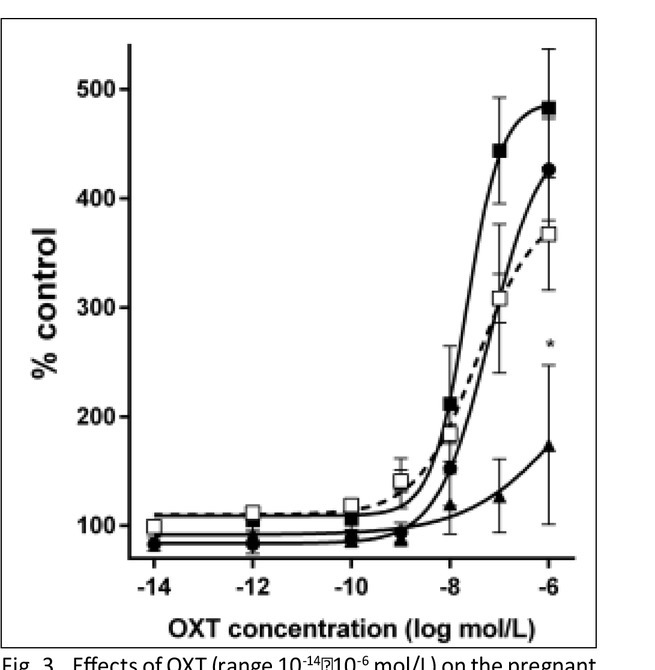
Effects of OXT (range 10^-14^–10^-6^ mol/L) on the pregnant myometrium, as measured by the area under the curve. (□) – OXT alone, (▪) – after preincubation with 3·10^-4^ mol/L L-NNA, (▴) – after incubation with 10^-4^ mol/L DETA/NO, (•) – after preincubation with 3·10^-4^ mol/L L-NNA followed by incubation of 10^-4^ mol/L DETA/NO. Each point represents the mean ± SEM of N =8 individual myometrial strips from different patients. Spontaneous contractions of the myometrial strips before OXT were treated as a control.

In the pregnant uterine strips, OXT (range 10^-14^-10^-6^ mol/L) caused a dose-dependent increase of AUC, as shown previously [18, 19] (Fig. 3). The presence of 10^-4^ mol/L DETA/NO caused a significant attenuation in the contractile response to OXT at concentrations higher than10^-8^ mol/L. Inhibition of NO synthesis alone or in combination with DETA/NO did not alter the OXT contractile effect in the concentration-response curve (Fig. 3).

Moreover, no significant variation in the mean value for log EC_50_ for OXT (range 10^-14^-10^-6^ mol/L) was observed between the group with OXT alone and other groups. E_max_ values for OXT after preincubation with NO donor were extensively lower than in the other three groups (p<0.05). In contrast, E_max_ values for OXT alone did not significantly differ compared to other groups (Tab. I).

**Table I:** Log EC_50_ and E_max_ for OXT (range 10^-14^ - 10^-6^ mol/L) on spontaneous contractions of the pregnant myometrium. The values are mean ± SEM of N individual myometrial strips from different patients. N=6 for all comparisons which were done in reference to control. *p<0.05 versus the Group 1; †p<0.05 versus the Group 3.

Groups	OXT	
	logEC 50	Emax
Group 1 (no blocker or NO donor - Control)	-7.61±0.18	367.30±51.53
Group 2 (preincubation with L-NNA)	-7.64±0.18	482.70±54.27
Group 3 (preincubation with DETA/NO)	-6.68±1.05	174.10±73.08*
Group 4 (preincubation with L-NNA + DETA/NO)	-6.97±0.39	426.60±46.88†

## Discussion

Inhibition of NOS significantly increased myometrial contractility, which was to be expected for tissue in which NOS activity is regulating muscle tension. This observation is consistent with the findings of Buhimschi *et al*., who reported that incubation with L-NAME increased the tension of rat myometrial strips in a concentration-dependent manner. [16]. Our previous report on human tissue was also consistent with the current results [14]. Also in a clinical study, Maciejewski et al. demonstrated that the decreased level of NO in patients with preterm labor may be associated with the initiation of uterine contraction before 37 weeks of gestation [7]. However, the results presented are in marked contrast with the findings of Bartlett *et al*. that eNOS, iNOS and nNOS proteins were not expressed at detectable levels in myocytes of the human myometrium at any stage of pregnancy. [20]

Nitric oxide donors cause attenuation of the myometrial contractility in humans [14,16] as well as in animals [9,21], which was also confirmed in our present study. Stronger inhibition after 20-hour incubation than after 20 min incubation seems to contrast with the release rate of the NO donor according to the decay law dx= -λxdt. It has been found that numerous substances that are administered are distributed and metabolized according to the exponential distribution [22]. Control pregnant uterine strips after 20-hour incubation showed a substantial decrease in spontaneous contractions. Increased sensitivity to the stimuli of the pregnant uterus may cause its different response to NO prolonged exposure.

Unpredicted outcome was our observation of a significant reduction in myometrium contractions after 20-minute incubation with DETA/NO after earlier inhibition of endogenous NO production by L-NNA compared to 20-minute incubation with DETA/NO alone. We anticipated to sum up the effect of endogenous and exogenous NO influence on uterine contractions. It has been confirmed, both by *in vitro* and *in vivo* studies that exogenous NO can decrease endogenous eNOS activity, regardless of the changes in gene expression [23]. However, our results demonstrated that when the functional balance between constrictor factors and endogenous NO is affected, exogenous NO appears to have a stronger impact on the attenuation of the uterine contractility. This effect can be explained by the predominance of the cGMP-independent pathway over the cGMP-dependent cascade when NOS is inhibited. Moreover, this excessive effect is much stronger [24]. It should also be taken into account that endogenously generated NO can have an inhibitory influence on both cGMP-dependent or –independent smooth muscle relaxation evoked by exogenous NO donors.

It is extensively acknowledged that the augmented production of NO by the pregnant myometrium serves to maintain relative uterine quiescence and a decrease of NO production close to term may be one of the triggering signals to start delivery activity. Changes in iNOS or cGMP expression have been examined in pregnant rats, guinea pigs, rabbits, and humans. Taken together, these studies support the hypothesis that endogenously produced NO *via* iNOS maintains relative myometrial quiescence during pregnancy, although the role of NO in the human myometrium has been questioned [20]. OXT has long been ascribed a major involvement in the mechanisms of labor, both in preterm and at term pregnancies. The present study showed that the continuous release of NO from its donor substantially decreased the response of human pregnant uterine strips to increasing concentrations of OXT.

In recent years, it has been hypothesized that NO-mediated relaxation is dependent on S-nitrosylation of specific and critical proteins involved in the relaxation of uterine smooth muscle [25]. It has been reported that prolonged exposure to DETA/NO of some lines of human cell culture results in dinitrogen trioxide (N_2_O_3_) formation and S-nitrosylation of key cellular proteins [26]. Therefore, it is possible that this process may result in contraction when considering the myosin light chain phosphatase. DETA/NO mediated cytotoxicity, through the action of N_2_O_3_ at high concentration or in the case of long-term exposure, can cause cell death. The above effects should be tested in experiments using N_2_O_3_ scavengers. On the other hand, the influence of N_2_O_3_ scavengers on smooth muscle contraction, as used by Ali et al., has not been tested, while for example morpholine (another N_2_O_3_ scavenger) exhibits vasodilator activity *in vitro* and *in vivo* [26, 27]. Thus, further research should be carried out to explain the mechanisms of both an additional DETA/NO inhibitory effect on spontaneous contractility and increased contractile response to OXT.

Prolonged exposure to DETA/NO may activate the MAPK/ERK signaling system and increase the production of interleukin 6 (IL-6) in skeletal myotubes, and IL-6 is a potent pro-inflammatory cytokine in human myometrium. IL-6 increases cyclooxygenase-2 and OXTR in cultured uterine smooth muscle cells but IL-1β initiates pregnant uterine contractility [28]. IL-6 initially causes contraction and then reduces cellular metabolic function with prolonged exposure [29]. Therefore, further immunohistochemical studies with blockers to the MAPK/ERK signaling system could explain both the additional inhibitory effect of DETA/NO on spontaneous contractility and the enhanced contractile response to OXT.

Current functional experiments with previous data [9, 16] support the possible application of NO donors as tocolytics in preterm labor. However, when NO synthesis was inhibited, the myometrium response to OXT did not change considerably, even in the presence of DETA/NO. This fact may indicate that the reaction of the pregnant myometrium to OXT changes only when endogenous NO is produced. In the animal model, administering the NOS-inhibiting agent virtually produced all the symptoms of preeclampsia, suggesting a direct relationship between NO production and vascular changes in a normal pregnancy [10,30]. Interactions leading to abnormal physiology and clinical syndrome related to preeclampsia are very complex and diverse combinations of prompting factors can lead to an equivalent dysfunctional mechanism. In the light of the data presented, the use of NO donors in the treatment of preterm labor should be questioned and requires further investigation.

Undoubtedly, problems and questions, like many other previous studies on the pathophysiology of preeclampsia, still remain to be elucidated. Nevertheless, our study is part of the abundance of research on the basic science and physiological changes in this syndrome. Based on this research, we provide evidence to support the hypothesis that a continuous supply of NO to the human pregnant myometrium environment attenuates its response to OXT, but only when endogenous NO production is not impaired. Complicated NO-mediated physiology involving NO requires advanced biochemical and biomolecular research that can support or counter both our findings and those reported above.
